# Flor Yeast Diversity and Dynamics in Biologically Aged Wines

**DOI:** 10.3389/fmicb.2018.02235

**Published:** 2018-09-25

**Authors:** Vanessa David-Vaizant, Hervé Alexandre

**Affiliations:** ^1^AgroSup Dijon, PAM UMR A 02.102, Université Bourgogne Franche-Comté, Dijon, France; ^2^Equipe VAlMiS, Institut Universitaire de la Vigne et du Vin, Dijon, France

**Keywords:** flor yeast, biofilm, wine, *Saccharomyces cerevisiae*, scanning electron microscopy, *FLO11*, vin jaune

## Abstract

Wine biological aging is characterized by the development of yeast strains that form a biofilm on the wine surface after alcoholic fermentation. These yeasts, known as flor yeasts, form a velum that protects the wine from oxidation during aging. Thirty-nine velums aged from 1 to 6 years were sampled from “Vin jaune” from two different cellars. We show for the first time that these velums possess various aspects in term of color and surface aspects. Surprisingly, the heterogeneous velums are mostly composed of one species, *S. cerevisiae*. Scanning electron microscope observations of these velums revealed unprecedented biofilm structures and various yeast morphologies formed by the sole *S. cerevisiae* species. Our results highlight that different strains of *Saccharomyces* are present in these velums. Unexpectedly, in the same velum, flor yeast strain succession occurred during aging, supporting the assumption that environmental changes are responsible for these shifts. Despite numerous sample wine analyses, very few flor yeasts could be isolated from wine following alcoholic fermentation, suggesting that flor yeast development results from the colonization of yeast present in the aging cellar. We analyzed the *FLO11* and *ICR1* sequence of different *S. cerevisiae* strains in order to understand how the same strain of *S. cerevisiae* could form various types of biofilm. Among the strains analyzed, some were heterozygote at the *FLO11* locus, while others presented two different alleles of *ICR1* (wild type and a 111 bp deletion). We could not find a strong link between strain genotypes and velum characteristics. The same strain in different wines could form a velum having very different characteristics, highlighting a matrix effect.

## Introduction

Wine is produced through the action of a complex microbial consortium (Petruzzi et al., 2017) composed among others of numerous non-Saccharomyces yeast species and a high diversity of *S. cerevisiae* (Capece et al., 2016). In the world of *S. cerevisiae*, flor yeast constitutes an exception. Flor yeast or flor velum yeasts can grow at the surface of different wines. These flor yeasts can be found in very specific wine processes known as biological aging practiced in Spain (Andalusia), Italy (Sardinia), Hungary and France (Jura) to produce Xeres, Vernaccia di Oristano, Szamorodni, and Vin Jaune wines, respectively.

In the classical wine process, yeast dies in the absence of sugar and oxygen at the end of the alcoholic fermentation. However, in the case of biological aging, the common characteristic of these wines is that after alcoholic fermentation, they are transferred into barrels, leaving an airspace. According to different authors (Esteve-Zarzoso et al., 2001; Aranda et al., 2002) in these conditions of nitrogen and sugar depletion encountered at the end of alcoholic fermentation, the yeast shifts from a fermentative to an oxidative metabolism favored by the presence of oxygen. Furthermore, at the diauxic shift, an increase in *FLO11* expression is observed which leads to an increase in cell surface hydrophobicity, facilitating the aggregation of cells and the entrapment of carbon dioxide, allowing the cell aggregate to rise to the surface and develop a biofilm (Zara et al., 2005). Thus, based on this model, yeasts responsible for alcoholic fermentation could be responsible for forming biofilm. However, to our knowledge, only two studies have compared the yeast *Saccharomyces* present during alcoholic fermentation to those present in the velum (Esteve-Zarzoso et al., 2001; Naumova et al., 2005). The first study concluded that yeasts responsible for alcoholic fermentation are different from velum yeast. However, the must in this study was inoculated with commercial yeast (Esteve-Zarzoso et al., 2001) which could have influenced the development of indigenous yeast. In the second study, Naumova et al. (2005) demonstrated that among all the *S. cerevisiae* yeasts isolated at distinct stages of sherry making (young wine, solera, and criadera) in various winemaking regions of Spain, that sherry yeasts diverged from primary winemaking yeasts. Thus, not all *Saccharomyces* are able to form a biofilm (Alexandre, 2013; Legras et al., 2016). The ability to form a biofilm is closely linked to the specific *FLO11* alleles present in flor yeast (Fidalgo et al., 2006). Indeed, the *FLO11* promoter is 0.1 kb shorter and the coding sequence is 1 kb larger in flor-forming yeast compared to non-flor-forming yeast (Fidalgo et al., 2006; Zara et al., 2009; Legras et al., 2014). These changes reflecting evolutionary adaptations (Fidalgo et al., 2006) result in increased protein glycosylation and hydrophobicity of the Flo11 glycoprotein of flor yeast (Zara et al., 2005; Fidalgo et al., 2006), which allows cells to form a biofilm. Other distinct genetic features characterize *S. cerevisiae* flor yeast, including chromosomal polymorphism and aneuploidy (Bakalinsky and Snow, 1990; Martinez et al., 1995; Ibeas and Jimenez, 1996; Guijo et al., 1997; Legras et al., 2014). All the flor strains share these specificities and can be clustered in the same group that has evolved to adapt to the specific wine surface niche encountered during wine biological aging (Legras et al., 2016).

Recently, genome sequencing and comparative genomic analysis of the three *S. cerevisiae* strains used for the production of sherry-type wines in Russia has been reported (Eldarov et al., 2018). They observed than gene polymorphism not only affect *FLO11*, but also genes involved in yeast morphology, carbohydrate metabolism, ion homeostasis, response to osmotic stress, lipid metabolism, DNA repair, cell wall biogenesis. Gene polymorphism in sherry strains is mainly due to SNP/InDel accumulation. These authors also report the presence of genes in the flor strain missing in the reference strain such as *MPR1* gene coding for *N*-acetyltransferase that is involved in oxidative stress tolerance via proline metabolism.

Besides these genetic specificities which explain the ability of the yeast to form biofilm, metabolomics and proteomic studies have revealed how these yeasts have adapted physiologically and metabolically to their environment (Moreno-García et al., 2014, 2015, 2017). For example flor yeast are resistant to ethanol and it has recently been shown that ethanol tolerance could partly be due to activation of genes related with the unfolded protein response (UPR) and its transcription factor Hac1p (Navarro-Tapia et al., 2016). The proteomic studies highlighted the overexpression of proteins involved in non-fermentable carbon uptake, glyoxylate and TCA cycle and cellular respiration in flor yeast under biofilm conditions compared to flor yeast grown in synthetic media rich in sugar (Moreno-García et al., 2015). Although the genetic, metabolic and physiological specificities that allow flor yeasts to develop as biofilms have been described and reviewed in-depth (Alexandre, 2013; Legras et al., 2016), knowledge on flor yeast ecology is scarce. The origin of these yeasts is still unknown, raising the question of whether they are present on grapes or found in the cellar? As stated above, there is still no strong evidence that yeasts responsible for alcoholic fermentation are the same as those present in the velum. It has been shown that *Brettanomyces bruxellensis* is sometimes present with *S. cerevisiae* (Ibeas et al., 1996); however, this has never been confirmed and it is still unknown whether other species are present in the velum. Charpentier et al. (2009) reported that different *S. cerevisiae* strains could be present in one velum but it is still unknown if this is a general feature. Furthermore, wine aging for Vin Jaune, Xeres, Vernaccia di Oristano and Szamorodni lasts several years (6 years for vin jaune). During these 6 years of aging, many changes can occur in the cellar environment, such as that of the temperature between summer and winter. To our knowledge, no study has yet been performed on these changes in environmental conditions responsible for flor yeast succession and community shift. The aim of the present study was to try to answer these questions.

## Materials and Methods

### Velum and Strain Isolation Protocol

All the microorganisms were isolated from French Vin Jaune originated from the Jura region (France) and made from Savagnin grape variety which come from two different wine estate working with indigenous yeasts. The Savagnin wines samples contained depending on the vintage 12.5–13.5% (v/v) alcohol, with a pH ranging from 3.1 to 3.4, and the acetic acid ranging from 200 to 500 mg/L.

Microorganisms were sampled from wine at the end of alcoholic fermentation, from wine at the beginning of the biological aging process (when the wine is transfer from fermentation tank to barrels for aging), and from velum. Thirty-seven velums were recovered by sliding stainless steel chips under the velum present at the surface of the wine. To isolate yeast and bacteria, serial dilutions were performed with each sample and each dilution was spread either on YPD medium (5 g.l^-1^ yeast extract, 10 g.l^-1^ peptone, 20 g.l^-1^ glucose, and 20 g.l^-1^ agar supplemented with chloramphenicol at 200 ppm to inhibit the development of bacteria) and LAC medium pH 5 (7.8% v/v white grape juice, 33 g.l^-1^ yeast extract, 0.6 g.l^-1^ tween 80, 80 mg.l^-1^ MnSO_4_ H_2_O, and 20 g.l^-1^ agar) supplemented with natacid at 50 ppm, respectively. Cultures were incubated for 48 h and 1 week at 25°C for yeast and bacteria respectively.

### Biological Aging With Isolated Strains

Five strains of *S. cerevisiae* isolated from five velums with different morphological characteristics (14.28O, 34.22O, 36.2J, 8.1J, 23.1O) were cultivated in 10 ml of YPD broth (36 h, 28°C). Each strain was grown in two different synthetic Fornachon media (Fornachon, 1953) containing either 4 or 10% (v/v) ethanol and two different French vin jaunes made from Savagnin grapes. Synthetic Fornachon medium was prepared as follows: yeast extract 1 g.l^-1^, (NH_4_)_2_SO_4_ 0.5 g.l^-1^, MgSO_4_ 1 g.l^-1^, CaCl_2_ 0.5 g.l^-1^, adjusted to pH 3.2, autoclaved for 20 min at 120°C, following the addition of 4% or 10% (v/v) ethanol. Each Savagnin wine and synthetic Fornachon medium was inoculated with 10^6^ cell.ml^-1^. Each velum was observed after 1 month. The identity of each strain from each velum was controlled by PCR interdelta analysis as described below.

### Scanning Electron Microscopy

Cells were fixed on stainless steel by a solution of 2.5% glutaraldehyde in 0.1M phosphate buffer pH 7.2 for 1 h at 4°C. The samples were then washed three times with phosphate buffer for 20 min at room temperature. Dehydration was performed by successive immersions in solutions of increasing ethanol content (70, 90, 100%), then three times for 10 min each in successive baths of ethanol-acetone solution (70:30, 50:50, 30:70, 100) and air-dried. Afterward, the samples were coated with a thin carbon layer using a CRESSINGTON 308R and observed with a JEOL JSM7600F scanning electron microscope (JEOL, Ltd.). Scanning electron microscopy was performed at 5 kV and the samples were observed at a working distance of 14.9 mm.

### Identification of Yeast Isolate

Genomic DNA of yeast was prepared from yeast cultures on YPD agar after 2 days of incubation with InstaGene Matrix Bio-Rad. First InstaGene Matrix had to be mixed at moderate speed on a magnetic stirrer to maintain the matrix in suspension then briefly pick an isolated yeast colony and suspend it in 30 μL of InstaGene Matrix. The suspension was incubated in a thermal cycler at 56°C for 50 min, then at 100°C for 8 min. Afterward, 30 μL DEPC water was added and the suspension centrifuged at 16,250 × *g* for 3 min. 3 μL of the resulting supernatant was used for 30 μL PCR reaction. For species identification, the 5.8S-ITS region was amplified by PCR with the primers ITS1 5′-TCCGTAGGTGAACCTGCGG-3′ and ITS4 5′-TCCTCCGCTTATTGATATGC-3′. PCR was performed in 40 μl of 1.5 mM MgCl_2_, 0.2 mM dNTPs, 1 μM of each primer, 0.025 U of *Taq* polymerase (Promega Corp., Madison, WI, United States) and 100 ng of yeast DNA. A T100 thermal cycler (Bio-Rad, Hercules, CA, United States) was used with a program described elsewhere (Esteve-Zarzoso et al., 1999). The amplified PCR products were analyzed by capillary electrophoresis on a MultiNA MCE 202 (Shimadzu, France) at 37°C for 75 s using the DNA-1000 kit (Shimadzu, France) containing the separation buffer with SyBer Gold (Invitrogen, France) and an internal size calibrator. They were automatically injected onto chips with a maximum rate voltage of 1.5 kV and a maximum current of 250 mA; the peaks were identified using a LED-excited (470 nm excitation wavelength) fluorescence detector. The size of the amplified DNA fragments was calculated on the MultiNA using the (GeneRuler 100 bp Plus DNA Ladder, Thermo Fisher Scientific, Inc., Waltham, MA, United States). Then the PCR products were sequenced with a cycle extension DNA sequencer (Beckman Coulter Cogenics, Essex, United Kingdom). The BLASTN algorithm was applied to the GenBank database for sequence identification^1^. All sequences are available on NCBI under accession numbers.

For *S. cerevisiae* differentiation, PCR interdelta analysis was performed according to Legras and Karst (2003) using *S. cerevisiae* DNA extracted as described below. One fresh colony was suspended in a microcentrifuge tube with 200 μL buffer (2% Triton X-100, SDS, 100 mM NaCl, 10 mM Tris-HCl, pH 8.0, 1 mM Na_2_ EDTA). 80 μL of chloroform-alcohol isoamylic (25:24:1) and 0.3 g glass beads (Sigma, Z250465) were added. The suspension was vortexed at the maximum setting for 2 min and place in ice for 2 min., 200 μL Tris-EDTA, pH 8.0, was added mixed to each tube and the suspension was centrifuged for 5 min at 12,500 rpm. The supernatant was transferred to a new microcentrifuge tube and 1 mL absolute ethanol was added to precipitate the DNA. The pellet was washed with 1 mL of 70% ethanol and centrifuged for 3 min at 12,500 rpm. The DNA pellet was dried at 90°C for 5 min and suspended in 50 μL of milliQ water. The DNA concentrations of the samples were then standardized (100 ng/μl) on the basis of optical density at 260 nm, by adding MilliQ water, as appropriate, and the samples were then stored at 4°C.

### Total DNA Extraction

At least 100 μL of velum were centrifuged for 5 min at 4°C, 12,000 rpm. The total DNA of pelleted microorganisms was extracted as described by Longin et al. (2016) using CTAB, proteinase K at 10 mg/mL and PVPP at 10%. The DNA concentrations of the samples were then standardized (100 ng/μl) on the basis of optical density at 260 nm, by adding DEPC-treated water, as appropriate, and the samples were then stored at -20°C.

### DGGE Analysis

The D1 domain of the fungal 26S rRNA gene was amplified with the primers NL1-GC (5′-CGCCCGCCGCGCGCGGCGGGCGGGGCGGGGGCCATATCAATAAG CGGAGGAAAAG-3′) and LS2 (5′-ATTCCCAAACAACTC GACTC-3′), as reported in a previous study (Cocolin et al., 2000). The NL1-GC primer had a 39-bp GC-clamp sequence at its 5′ end to prevent the complete denaturation of amplicons. PCR was performed in a reaction volume of 50 μl, with 1.5 mM MgCl_2_, 0.2 mM dNTPs, 0.2 μM of each primer, 2.5 U of *Taq* polymerase (Promega Corp., Madison, WI, United States) and 10–100 ng of yeast DNA. Reactions were run for 30 cycles of denaturation at 95°C for 60 s, annealing at 52°C for 45 s and extension at 72°C for 60 s. An initial 5-min denaturation at 95°C and a final 7-min extension at 72°C were used. The products (250 bp) were analyzed by capillary electrophoresis on a MultiNA MCE 202 (Shimadzu, France).

Vertical polyacrylamide gels (acrylamide-bis acrylamide 37, 5:1, Bio-Rad, Hercules, CA, United States), with a denaturing gel of 35–50% polyacrylamide, were used for DGGE. The 100% chemical denaturing solution consisted of 3.5 M urea (Sigma-Aldrich) and 20% (v/v) formamide (Sigma-Aldrich) in 2 mL TAE (50×).

APS (Sigma-Aldrich A3678) and TEMED (Sigma-Aldrich T9281) were added to each gel before being mixed at 4°C to create the denaturing gradient. We mixed 40-μl samples of PCR amplicons with 10 μl of (100%) glycerol before loading on the gel. A DCode apparatus (Bio-Rad) was used for DGGE in 1 × TAE, at 60°C for 5 h 30 min, with a constant voltage of 130 V. The gels were stained 10 min with 10 × BET (Sigma-Aldrich E1510) in 1 × TAE and the bands were visualized and photographed under UV transillumination. The bands were excised from the gels and the DNA was eluted overnight in 40 μl of water MilliQ at 4°C. The DNA was re-amplified with the same pair of primers without the GC-clamp and sequenced with a cycle extension DNA sequencer (Beckmann Coulter Cogenics, Essex, United Kingdom). The BLASTN algorithm was applied to the GenBank database for sequence identification (see footnote 1). All sequences are available on NCBI under the following accession number from MH276962 to MH276980 and from MH252537 to MH252566.

### *FLO11* Polymorphisms

The length of FLO11p was measured from the amplification of *FLO11* alleles with the primers *FLO11* (Flo11IntFw CTCCCTCATCATGTTGTGGTTC), and (Flo11IntRv AACGACGGTGGTTGAGACAA) according to Legras et al. (2014). The PCR reaction was performed with Expand High Fidelity DNA polymerase (Roche) to amplify this long DNA fragment. The PCR program: 94°C for 2 min, followed by 10 cycles: 94°C for 15 s, 61°C for 30 s, 68°C for 5 min and 20 cycles at 94°C for 15 s, 61°C for 30 s, and 68°C for 5 min + a 5 s cycle prolongation for each successive cycle. The PCR products were subjected to electrophoresis for 1 h at 100 V in 0.7% agarose gels which were then stained with ethidium bromide (14 mg/ml) for visualization of the DNA bands under UV light. Fragment sizes were estimated by comparison with DNA size markers (GeneRuler 1 Kb DNA Ladder, Thermo Fisher Scientific, Inc., Waltham, MA, United States), with Quantity One 4.6.5 software from Bio-Rad.

The *FLO11* promoter deletion was performed with the primer pair Flo11promFw CAGCCCCAGAGTATGTTCTCACAG and Flo11promRv AATCACCTTCTAAACGCTCGGA. This PCR was performed with *Taq* polymerase (Promega Corp., Madison, WI, United States). The PCR program was 95°C for 5 min, followed by 30 cycles of 95°C for 30 s, 56°C for 45 s, and 72°C for 1 min. The presence of the deletion was detected by capillary electrophoresis on a MultiNA MCE 202 (Shimadzu, France).

### Cluster Analysis of the Strains

The inter delta sequence patterns obtained after capillary electrophoresis were used to construct a presence/absence matrix, taking into account the total number of different bands observed. All visible bands were assigned a number based on relative position to the DNA ladder. Each position was then assigned a “0” or a “1” to indicate the absence or presence of the band, respectively. Then, 0/1 matrix was used to generate a dissimilarity dendrogram based on the Dice coefficient using the UPGMA algorithm with XLstat (Addinsoft, Inc.).

## Results and Discussion

Flor yeasts have been extensively studied from the genomic, proteomic, and metabolomics angles (Alexandre, 2013; Legras et al., 2016), because they provide an interesting biological model for studying the adaptation of yeasts to a specific niche. However, little information exists on the dynamics of yeast from alcoholic fermentation until the end of the biological aging process. Jura “vin jaune” is a sherry like wine whose aging lasts 6 years in partly filled barrels that allow a velum yeast to develop. The objective of the present study was to investigate the nature of the yeast present at the end of alcoholic fermentation until the end of the 6-year aging process.

### Flor Yeast Velum Characteristics

**FIGURE 1:**
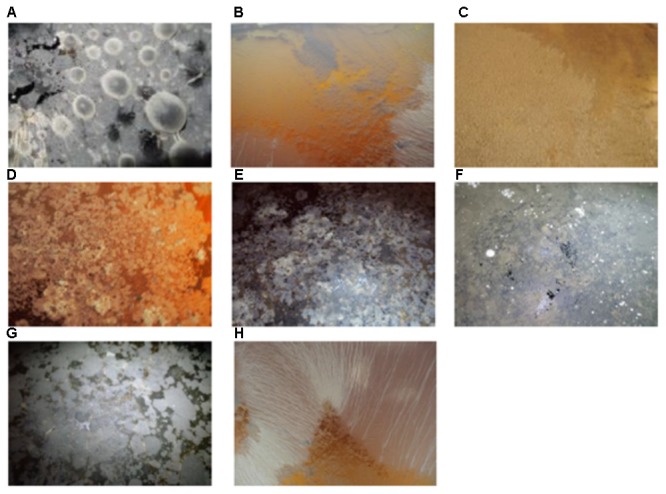
Flor yeast velum characteristic: **(A)** gray velum with blisters; **(B)** pink velum with wrinkles; **(C)** yellow thick and granular velum; **(D)** mix of white, brown and yellow velum; **(E)** mix of gray, black, yellow and white velum; **(F)** gray fine and smoothy velum; **(G)** black velum with islands; **(H)** white velum with wrinkles.

Two thousand five hundred and sixty-five yeast strains were isolated from two different cellars in Jura vineyard (France) from 41 Savagnin Jura wines from the 2007 to 2013 vintages. Sampling was done in winter, summer, and autumn. Several examples of the nature of the velum are given **Figure 1**. Surprisingly, this is the first morphological characterization of velum. Indeed, despite the numerous studies on flor yeast, there are no detailed descriptions of the velum present on wines. **Figure 1** shows the extraordinary diverse nature of the velum found. White, cream, yellow, pink, deep gray, brown and black velums were observed. These velums were not homogenous, and wines could be completely covered **Figures 1C,F** or partly covered by velum (**Figures 1A,B,D–F**). This might reflect the age of the velum. When the velum starts to grow, it forms a small island on the surface of the wine (**Figure 1G**) and then expands to cover the entire surface of the wine (**Figure 1F**). Different velum morphologies could also be observed, some appear smooth (**Figure 1F**), others are granular (**Figure 1C**), while others present wrinkles (ruffled pattern) (**Figures 1B,H**).

**Figure 1A** presents kinds of blisters which might reflect the effect of carbon dioxide entrapped in the velum. Another characteristic of this velum is the presence of different colors which might reflect the presence of different microorganisms. For example, in **Figure 1F** white spots are present on the deep gray velum; in **Figure 1E** a mix of gray, black yellow and white can be seen while **Figure 1D** is characterized by a mix of white, brown, and yellow. Thus, our study revealed that contrary to what is reported in the literature (Charpentier et al., 2009) velums are not only gray or white.

### Flor Yeast Species Identification

Microorganisms present in the velum were isolated either on bacteria or yeast medium. No bacteria could be recovered from any of the velums sampled. Two thousand five hundred and sixty-five yeasts were isolated from these velums to identify the nature of the species present. However, despite the use of different media, some of the isolates were not able to recover growth. For these reasons, DGGE was used to identify the species present in the velums. **Table 1** groups the identification of the species present in wine at the end of alcoholic fermentation, after the transfer of the wine into barrels for biological aging, and in velum. Four different Savagnin Jura wines were sampled: three in wine estate A and one in wine estate B. As expected, at the end of alcoholic fermentation, 100% of the yeast present belonged to *S. cerevisiae* species (W1AF, W2AF W3AF, W18AF) (**Table 1**). After the transfer of the wine from tanks to barrels for biological aging, different species could be found, as shown by PCR-ITS and DGGE. As shown in **Table 1** most of the yeasts present in the velum belonged to *S. cerevisiae* species, which support previous reports (Martinez et al., 1995; Ibeas et al., 1997; Charpentier et al., 2009; Pozo-Bayón and Moreno-Arribas, 2011). However, in rare cases different species could be identified in the same velum (**Table 1**). Velum V35 and V37 was composed of both *S. cerevisiae* and *Dekkera bruxellensis* while Velum V12 was formed with *Zygosaccharomyces lentus* together with *S. cerevisiae*. These results demonstrate that other wine yeast species can form biofilms and survive this harsh environment. The presence of such species that reflect wine alteration has already been reported (Ibeas et al., 1996; Suarez-Lepez and Inigo-Leal, 2004). The most important aspect of this study is that although most velums were composed of *S. cerevisiae*, they presented very different velum characteristics. This means that a single species can lead to different types of velum in terms of color, structure and surface characteristic. To explain these unexpected observations, we investigated the nature of the differences in velum morphology using scanning electron microscopy.

**Table 1:** Identification of wine and velum yeast by different molecular techniques (5,8S PCR and 26S DGGE).

Wine estates	Sample wine velum	PCR ITS 5,8S	DGGE 26S
A	W1AF	*Saccharomyces cerevisiae*	
	W1	*Zygosaccharomyces bailii*	
		*Saccharomyces cerevisiae*	Ascomycota
		*Debaryomyces carsonii*	*Kregervanrija fluxuum*
		*Zygosaccharomyces lentus*	*Debaryomyces* sp.
		*Kregervanrija fluxuum*	
A	W2AF	*Saccharomyces cerevisiae*	
	W2	*Pichia membranefaciens*	*/*
		*Saccharomyces cerevisiae*	
		*Dekkera bruxellensis*	
A	W3AF	*Saccharomyces cerevisiae*	
	W3	*Saccharomyces cerevisiae*	*Pichia membranefaciens*
		*Pichia membranefaciens*	
		*Dekkera bruxellensis*	
A	V4	*Saccharomyces cerevisiae*	*/*
A	V5	*Saccharomyces cerevisiae*	*Saccharomyces cerevisiae*
A	V6	*Saccharomyces cerevisiae*	*/*
A	V7	*NC*	*/*
A	V8	*Saccharomyces cerevisiae*	*Saccharomyces cerevisiae*
A	V9	*NC*	*/*
A	V10	*NC*	*/*
A	V11	*Saccharomyces cerevisiae*	*/*
A	V12	*Zygosaccharomyces lentus*	*Saccharomyces cerevisiae*
		*Saccharomyces cerevisiae*	
A	V13	*NC*	*Saccharomyces cerevisiae*
A	V14	*Saccharomyces cerevisiae*	*/*
A	V15	*Saccharomyces cerevisiae*	*/*
A	V16	*Saccharomyces cerevisiae*	*/*
A	V17	*Saccharomyces cerevisiae*	*Saccharomyces cerevisiae*
B	W18AF	*Saccharomyces cerevisiae*	
	W18	*Saccharomyces cerevisiae*	*Saccharomyces cerevisiae*
		*Dekkera bruxellensis*	*Dekkera bruxellensis*
B	V19	*Saccharomyces cerevisiae*	*Saccharomyces cerevisiae*
B	V20	*Saccharomyces cerevisiae*	*/*
B	V21	*Saccharomyces cerevisiae*	Ascomycota^∗^
B	V22	*Saccharomyces cerevisiae*	Ascomycota^∗^
B	V23	*Saccharomyces cerevisiae*	Ascomycota^∗^
B	V24	*Saccharomyces cerevisiae*	/
B	V25	*Saccharomyces cerevisiae*	/
B	V26	*Candida fermenticarens*	
		*Pichia anomala*	*Pichia anomala*
		*Pichia farinosa*	*Pichia farinosa*
		*Metschnikowia pulcherrima*	*Candida fermenticarens*
		*Saccharomyces cerevisiae*	Ascomycota^∗^
B	V27	*Saccharomyces cerevisiae*	*Saccharomyces cerevisiae*
B	V28	*Saccharomyces cerevisiae*	*Saccharomyces cerevisiae*
B	V29	*Saccharomyces cerevisiae*	*/*
B	V30	*Saccharomyces cerevisiae*	*Saccharomyces cerevisiae*
B	V31	*Saccharomyces cerevisiae*	*Saccharomyces cerevisiae*
B	V32	*NC*	*/*
B	V33	*Saccharomyces cerevisiae*	*/*
B	V34	*Saccharomyces cerevisiae*	*Saccharomyces cerevisiae*
B	V35	*Saccharomyces cerevisiae*	*Dekkera bruxellensis*
		*Dekkera bruxellensis*	
B	V36	*Saccharomyces cerevisiae*	*Saccharomyces cerevisiae*
B	V37	*Saccharomyces cerevisiae*	*Saccharomyces cerevisiae*
		*Dekkera bruxellensis*	*Dekkera bruxellensis*
B	V38	*Saccharomyces cerevisiae*	*Saccharomyces cerevisiae*
B	V39	*NC*	*Saccharomyces cerevisiae*
B	V40	*NC*	*Saccharomyces cerevisiae*
B	V41	*NC*	*Saccharomyces cerevisiae*

*WAF: strains isolated in wine at the end of the alcoholic fermentation. W: strain isolated in the wine at the beginning of the biological aging process. V: strain isolated in the velum. NC: not cultivable. ^∗^Ascomycota: Unidentifiable yeast, 100% similar by blast for V21-V22-V23-V26-W1 26S ribosomial RNA gene.*

### Scanning Electron Microscopy of Velum

**FIGURE 2:**
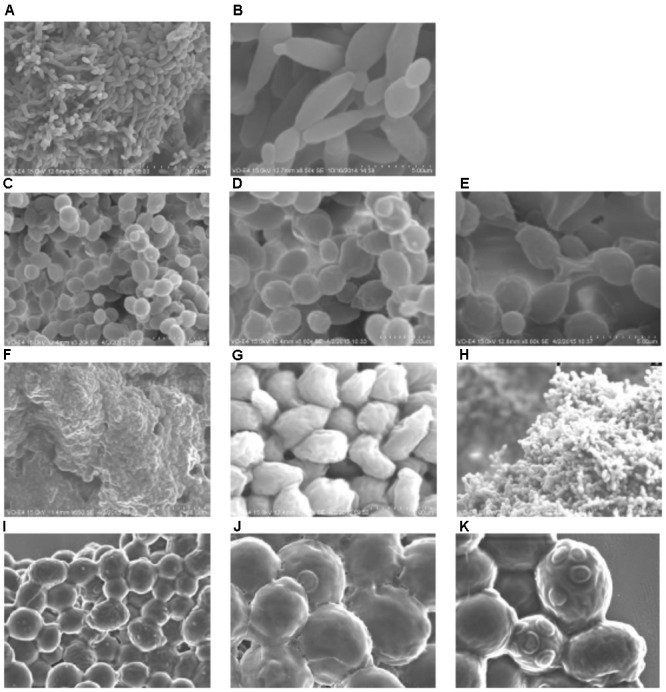
Scanning electron microscopy of velum: **(A)** ×2,000 **(B)** ×8,500 ovoid yeast and elongated pseudomycelium yeast; **(C)** ×3,000 **(D)** ×6,000 **(E)** ×8,000 yeast biofilm where yeasts are present in short chains of several cells; **(F)** ×650 **(G)** ×7,000 **(I)** ×5,000 **(J,K)** ×10,000 very dense biofilm with network of yeast, with all yeast cells embedded in an extracellular matrix; **(H)** ×1,000 velum in 3D like structure is visible formed by a sequence of ovoid cells attached together by their pole in an apparently disorganized manner.

Surprising images were obtained, highlighting huge differences between the biofilm structures of velums (**Figure 2**). Microscopic observations revealed distinct yeast morphologies (**Figure 2A**). Typical yeast shape-like cells (ovoid) together with elongated yeast could be observed (**Figures 2A,B**). This velum is composed of both *S. cerevisiae* and *Dekkera bruxellensis* and identified by the label ITS-RFLP (**Table 1**). Many different yeast morphologies were observed in the different velums under study. **Figures 2C–E** show a yeast biofilm in which yeasts are present in short chains of several cells. Yeast cells are recovered by an extracellular matrix (**Figure 2D**) and connected by an extracellular material (**Figure 2E**). This biofilm characteristic has already been observed previously (Zara et al., 2009). However, a third type of velum biofilm never reported before is presented (**Figures 2F,G,I–K**). A very dense network of yeast is observed for this biofilm, with all the yeast cells embedded in an extracellular matrix. Finally, **Figure 2H** shows another model of biofilm in which a 3D like structure is visible, formed by a sequence of ovoid cells attached together by their pole in an apparently disorganized manner. Our results show that there are extensive phenotypic variations between yeasts regarding biofilm morphology though they all belong to *S. cerevisiae* species except the biofilm shown in **Figures 2A,B**.

Variations in ploidy have been reported to play a key role in biofilm phenotypes (Hope and Dunham, 2014) and might explain the differences observed. However, according to Legras et al. (2014) most of the flor strains (70 flor strains studied from different countries) are diploid, which does not support the idea that biofilm phenotypic differences are linked to ploidy. On the other hand, aneuploidies are considered to be a potential mechanism allowing adaptation to flor aging (Guijo et al., 1997; Infante et al., 2003), but according to Legras et al. (2014) there is no substantial aneuploidy in flor yeast.

Differences in velum characteristics might be linked to different *S. cerevisiae* strains, therefore all the *S. cerevisiae* strains sampled from wines (at the end of alcoholic fermentation before velum formation), at the beginning of the biological aging process and velum formation were genotyped.

### Flor Yeast Strain Genotyping

**FIGURE 3:**
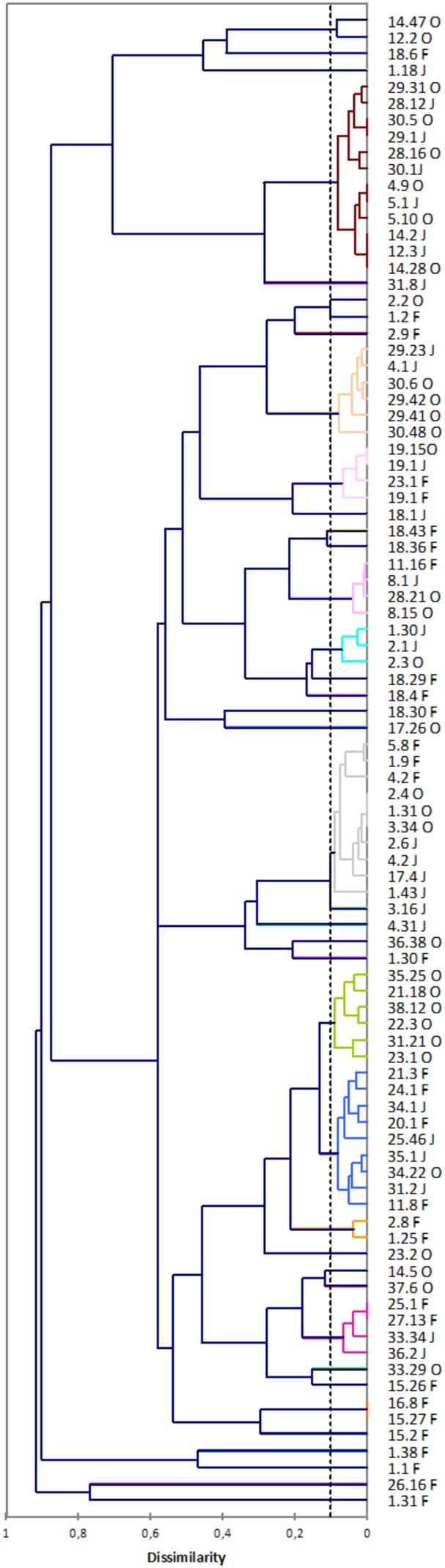
Dendrogram derived from UPGMA cluster analysis of inter delta using dissimilarity coefficients.

Among the 2,025 isolates belonging to *S. cerevisiae* determined by ITS RFLP, we found 69 different genotypes determined by subjecting inter delta data to clustering analysis (**Figure 3**). Among these 69 different *S. cerevisiae* strains, 38 of them were velum yeasts according to their ITS RFLP profile (**Table 2**). Indeed, in velum yeast an insertion in the ITS1 region led to an additional *Hae*III site which allowed differentiating flor-*S. cerevisiae* from classical *S. cerevisiae* (Esteve-Zarzoso et al., 2004; Charpentier et al., 2009). It is noteworthy that none of the *S. cerevisiae* strains isolated at the end of the alcoholic fermentation (samples W1AF, W2AF, W3 AF, W18 AF) gave the specific flor-*Saccharomyces cerevisiae Hae*III restriction pattern (**Table 2**). These results underline that the model proposed by Zara et al. (2005) might not be the general rule. Indeed, in their model, they propose that at the end of alcoholic fermentation, an increase of *FLO11* expression following diauxic shift leads to an increase of cell surface hydrophobicity and consequently favors cell aggregation and biofilm formation. However, as shown here and as far as we know, flor yeasts have never been isolated in wine at the end of the alcoholic fermentation.

**Table 2:** Identification of all the *Saccharomyces cerevisiae* strains isolated in all the velum according to their ITS RFLP profile.

Wine estates	Sample wine velum	*HaeIII* restriction PCR ITS	
		Velum	Fermentation
		*S. cerevisiae* strains	*S. cerevisiae* strains
A	W1AF		S31f, S1f, S3f, S8f, S1Of, S11f, S12f, S24f, S26f
	Wl	S1v, S8v, S11v, S12v, S38v	S13f, S14f, S15f, S31f
A	W2AF		S31f, S1f, S2f, S3f, S5f, S6f, S7f, S8f, S16f, S23f, S25f, S27f
	W2	S1v, S8v, S11v, S12v, S38v	
A	W3AF		S31f, S13f, S3f, S4f, S16f, S17f, S18f, S19f, S20f, S21f, S22f, S25f
	W3	S1v, S8v, S11v, S12v, S38v	
A	V4	S1v, S2v, S5v, S32v	
A	V5	S1v, S2v	
A	V6	S2v	
A	V7	/	
A	V8	S6v	
A	V9	/	
A	V10	/	
A	V11	S3v, S6v	
A	V12	S2v	S9f
A	V13	/	
A	V14	S2v, S26v	S9f
A	V15	S10v, S17v, S37v	
A	V16	S10v	
A	V17	S1v, S27v	
B	W18AF		S28f, S29f
	W18	S18v, S19v, S20v, S21v, S22v, S23v, S36v	
B	V19	S7v	
B	V20	S3v	
B	V21	S3v, S4v	
B	V22	S34v	
B	V23	S7v, S28v, S34v	
B	V24	S3v	
B	V25	S3v, S4v	
B	V26	S24v	
B	V27	S4v	
B	V28	S2v, S6v	
B	V29	S2v, S5v, S35v	
B	V30	S2v, S5v, S35v	
B	V31	S3v, S33v, S34v	
B	V32	/	
B	V33	S4v, S29v	
B	V34	S3v	
B	V35	S3v, S24v	
B	V36	S4v, S30v	
B	V37	S25v	
B	V38	S34v	
B	V39	/	
B	V40	/	
B	V41	/	

*All strains presenting the typical *HaeIII* velum yeast pattern are coded (Sv), the others Sf (for fermentation strain).*

The second analysis of yeast strains present in wine was performed after the transfer of the wine into barrels, at the beginning of the biological aging process. While there was still no visible velum at that stage, most of the yeast strains isolated belonged to *S. cerevisiae* species and most were flor-*Saccharomyces* yeasts (samples W1, W2, W3, W18) (**Table 2**). One exception could be observed in wine 1, where classical *S. cerevisiae* belonging to four different phenotypes were isolated (S13f, S14f, S15f, and S31f). This was not unexpected, since residual fermentation yeasts were present in wine during wine transfer. However, our results support the view that velum formation is due more to the implantation of flor yeast present in the cellar, barrels and materials. Indeed, ITS-RFLP profiles determined on all the *S. cerevisiae* strains isolated from velum (samples V4 to V41) demonstrated that except one strain (S9f) they all shared the common *Hae*III restriction pattern of flor yeast (**Table 2**).

### Dynamics of *Saccharomyces* Genotypes During the Biological Process Aging

To determine seasonal effects, the time of possible community shifts during aging, and the presence of any dominant genotype, we analyzed the frequency (%) of all the *S. cerevisiae* genotypes according to the wine estate, the vintage, and the seasons in wines at the beginning of the aging process and in velums (**Supplementary Table S1**). For ease of reading, data where extracted from this important table to present data in **Tables 3, 4**.

**Table 3:** Example of the distribution (%) of different *Saccharomyces cerevisiae* genotypes present in velum (V) showing that several different *Saccharomyces cerevisiae* strains could be present in a same velum.

	Velum									
		V4	V11	V14	V15	V23	V28	V29	V30	V31
*Saccharomyces cerevisiae* genotype	S1v	88								
	S2v			81			71	90	75	
	S3v		34							84
	S5v	8						10		
	S6v		66				29		25	
	S9f			13						
	S26v			6						
	S28v					50				
	S32v	4								
	S33v									16
	S34v					50				

**Table 4:** Example of the distribution (%) of different *Saccharomyces cerevisiae* genotypes present in velum (V) showing the shift from one strain to another according to the season.

			*Saccharomyces cerevisiae* genotype														
		Season	Slv	S2v	S8v	S28v	S24v	S3v	S6v	S27v	S7v	S34v	S29v	S4v	S5v	S33v	S30v
Velum	V5	W	100														
		S		100													
		A		100													
	V23	W			100												
		A				50	50										
	V12	S						34	66								
		A		100													
	V17	S	100														
		A								100							
	V23	W									100						
		A				50						50					
	V25	W												100			
		S						100									
	V31	S						84								16	
		A										100					
	V33	S												100			
		A											100				
	V35	S						100									
		A										100					
	V36	S												100			
		A															100

*W, winter; S, summer; A, autumn.*

**Table 3** clearly shows that velum could be formed by several different *S. cerevisiae* strains. For example velums V4, V11, V14, V15, V23, V28, V29, V30, and V31 possess two or three different *S. cerevisiae* strains. It was very puzzling to observe a shift from one strain to another during aging (**Table 4**). Indeed, velum V5 was composed of only one *S. cerevisiae* strain (S1v) for the sampling done in winter which was different from the strain genotype (S2v) present in the same velum in summer and autumn (**Table 4**). The same behavior was observed for V23, V25. The same observations were made when comparing summer and autumn periods for velums V12, V17, V31, V33, V35, and V36 (**Table 4**). These results suggest that some flor yeasts are better adapted than others and could competitively displace them. This dynamic nature of *S. cerevisiae* populations has been observed during alcoholic fermentation (Frezier and Dubourdieu, 1992; Versavaud et al., 1995; Schuller et al., 2005) but never reported for flor yeast.

This *S. cerevisiae* dynamic might be explained by changes in environmental conditions during the four seasons such as temperature (5–10°C in the cellar in winter and 25–30°C in the cellar in summer: cellars for aging are under the roof) (Charpentier et al., 2002). Indeed, this succession revealed that environmental conditions drive community shifts. Some strains of *S. cerevisiae* may be better adapted to higher temperatures than others, explaining their occurrence during summer, for example. During aging, the velum can sink in the wine because the cells are not adapted to the medium and changing environmental conditions which allow a better adapted strain to colonize the medium and form a new velum composed of a different strain. Other original information revealed by our study is that the same strain could be observed in velums from different wines. For example, the genotype profile S3v (**Supplementary Table S1**) was found in velum from vintages 2008, 2010, 2011, 2012 and the genotype profile S2v (**Supplementary Table S1**) was found in velum from vintages 2009, 2011, 2012. We could also observe patterns of sporadic presence, absence and reoccurrence. Profile S7v was absent from vintage 2008, appeared for the first time in 2010, though was absent from the 2011 velum and then reappeared in 2012. A similar dynamic pattern could be observed for profile S2v. These results reflect that there are a few dominant strains that are better adapted to the wine than others. Another surprising observation was the fact that the same *S. cerevisiae* genotype profile was found in velums with very different surface characteristics (**Table 5**). For example, *S. cerevisiae* profile S3v was found in velum with yellow (V21), white (V20, V25, V36), cream (V11), and brown colors and with different structures. One explanation could be that color reflects the evolution of the velum linked both to aging and wine composition. Indeed, during aging wine phenolic compounds oxidized (Danilewicz, 2012) and could be adsorbed by yeast cell walls which would stain the cells (Vasserot et al., 1997). Depending on wine composition, this oxidation might be more or less considerable, which could explain the color nuances from white to yellow and brown. Regarding velum structure differences, these could reflect differences in cell density, wine movement inside the barrels due to changes of the atmospheric pressure and Brownian movements due to temperature variation. However, strains isolated in thick white, yellow or brown velum were never isolated in thin gray or black velums which confirms previous results (Charpentier et al., 2009).

**Table 5:** Example of different *Saccharomyces cerevisiae* genotypes isolated in velum with different colors.

	*Saccharomyces cerevisiae* genotypes							
Velum color	S2v	S3v	S4v	S5v	S6v	S7v	S24v	S34v
White	X	X	X	X				X
V4, V5, V20, V24, V25,V31, V35, V36								
Gray	X		X		X	X		X
V6, V12, V19, V14, V23,V27, V33, V8, V22								
Cream	X	X		X		X		
V11, V28, V29, V30, V34								
Brown		X						
V34								
Yellow		X						X
V21								
Black							X	
V26								

**Supplementary Table S1** also reveals that although very rare, one strain that did not present the typical *HaeIII* (S9f) profile was present at 100% and 12% in two different velums from 2009 and 2011, respectively (**Table 2**). Such observations were reported before in Jura flor yeast (Charpentier et al., 2009).

All flor yeasts were isolated from two different wine estates 54 km away from each other. It is noteworthy that they did not share any common *S. cerevisiae* flor yeast. This result supports the existence of the geographic distribution of yeast profiles observed previously (Charpentier et al., 2009).

### Phenotype and Genotype Correlation

We determined both the size of the *FLO11* gene and IRC1 region of 55 *Saccharomyces* clones corresponding to 38 different strains according to interdelta profiles (**Table 6**). The amplification of a short sequence of ICR1 ncRNA for all flor-*Saccharomyces* gave two different sizes, 447 bp and 350 bp, corresponding respectively to the wild and 111 bp deletion of the ICR1 sequence, as previously reported (Fidalgo et al., 2006). Interestingly, some strains carried a wild and a deleted allele. On 52 isolates, 12 possessed both alleles, 19 possessed only the allele with the deletion in the ICR1ncRNA region and 21 possessed only the full length ICR1ncRNA allele (**Table 6**). The presence of both alleles in flor yeast has already been reported for Hungary isolated flor yeast (Legras et al., 2014). However, contrary to our observations, the authors did not find either allele in any of the Jura flor yeast isolates. The length of the core region of *FLO11* gene was sequenced for all the isolates and its size varied from 2.8 to 6.2 kb. These results agree with a previous report (Legras et al., 2014). Most of our isolates possessed a *FLO11* sequence longer than the sequence of wine yeast whose average size was 2.9 kb using the same primers (Legras et al., 2014). Regarding the promoter region, many isolates were heterozygote at the *FLO11* locus, which is in line with previous reports (Legras et al., 2014). It is noteworthy that different isolates that had been characterized as being the same yeast strain based on interdelta PCR analysis, could have different *FLO11* promoters and lengths (**Table 6**). Indeed, not all the clones (14.2J, 12.3J, 5.1J, 14.28O, 29.31O, 30.5O, 28.12J) sharing the profile S2v had the same *FLO11* promoter and/or ORF length (**Table 6**). The same phenomenon could be observed for clones sharing the same interdelta profile S1v.

**Table 6:** *FLO11* promoter size and ORF length variations, velum color and thickness of various flor strains [55 clones belonging to 38 genotypes (S1v to S38v)].

Velum yeast	Strain	*FLO11* diversity		Velum color	Velum thickness	
		Promoter	*FLO11*			
Lab reference	*S288c*	447 bp	3,300 bp			
	*5.8F*	447 bp	3,500 bp	White		Fine
	*1.43J*	447 bp	3,500 bp		nv	
	*2.4O*	447 bp	3,500 bp		nv	
S1v	*4.2J*	447 bp + 350 bp	3,500 bp	White		Fine
	*17.4J*	447 bp + 350 bp	3,400 bp + 4,100 bp	Cream		Fine
	*2.6J*	447 bp + 350 bp	3,400 bp + 4,100 bp		nv	
	*3.16J*	447 bp + 350 bp	3,400 bp + 4,200 bp		nv	

	*14.2J*	350 bp	3,500 bp	Gray		Fine
	*12.3J*	350 bp	3,500 bp	Gray		Fine
	*5.1J*	447 bp + 350 bp	6,200 bp	White		Fine
S2v	*14.28O*	447 bp + 350 bp	6,200 bp	Black		Fine
	*29.31O*	447 bp + 350 bp	6,200 bp	Cream		Thick
	*30.5O*	447 bp + 350 bp	6,200 bp	Cream		Thick
	*28.12J*	447 bp + 350 bp	6,200 bp	White		Fine

	*34.22O*	350 bp	3,500 bp	Brown		/
	*11.8F*	350 bp	4,500 bp	Cream		Thick
S3v	*20.1F*	350 bp	4,500 bp	White		Thick
	*24.1F*	350 bp	5,000 bp	White		/
	*21.3F*	350 bp	4,500 bp	Yellow		Thick

	*33.34J*	350 bp	3,500 bp	Gray		Thick
S4v	*36.2J*	350 bp	3,700 bp	White		Thick
	*27.13F*	350 bp	3,500 bp	Gray		Fine

S5v	*4.1J*	447 bp + 350 bp	3,300 bp	Cream		Fine

S6v	*8.1J*	447 bp	3,600 bp	Gray		Fine
	11.16F	447 bp	3,600 bp	Cream		Thick

S7v	*19.1J*	350 bp	3,700 bp	Gray		Fine

S8v	*1.25F*	447 bp	3,500 bp		nv	

S9f	*12.2O*	447 bp	2,300 bp + 2,900 bp	Gray		Fine
	*14.47O*	447 bp	2,300 bp + 2,900 bp	Black		Fine

S10v	*15.27F*	350 bp	4,200 bp	White		Thick

S11v	*2.2O*	447 bp	4,800 bp		nv	

	*2.3O*	447 bp	3,500 bp		nv	
S12v	*2.1J*	447 bp	3,700 bp		nv	
	*1.30J*	447 bp	3,500 bp + 4,700 bp		nv	
	*2.1J*	447 bp	3,700 bp		nv	

S16v	*1.30F*	447 bp	4,200 bp		nv	

S17v	*15.26F*	340 bp	3,500 bp	White		Thick

S18v	*18.4F*	447 bp	3,500 bp + 4,200 bp		nv	

S19v	*18.6F*	447 bp	4,200 bp		nv	

S20v	*18.29F*	447 bp	3,500 bp		nv	

S21v	*18.30F*	447 bp	4,000 bp + 4,200 bp		nv	

S22v	*18.36F*	447 bp	3,400 bp + 4,200 bp		nv	

S23v	*18.43F*	447 bp	3,400 bp + 4,200 bp		nv	

S25v	*37.6O*	340 bp	4,200 bp + 4,300 bp	White		/

S26v	*14.5O*	340 bp	4,200 bp + 4,300 bp	Gray		Fine

S27v	*17.26O*	447 bp + 350 bp	3,200 bp + 4,500 bp	Cream		Thick

S28v	*23.2O*	340 bp	2,800 bp	Gray		Fine

S29v	*33.29O*	340 bp	3,200 bp	Gray		Thick

S30v	*36.38O*	447 bp	3,100 bp + 3,500 bp	White		Thick

S32v	*4.31J*	447 bp + 350 bp	3,400 bp + 4,100 bp	White		/
	*23.1O*	350 bp	4,500 + 4,700 bp	Gray		Fine

S34v	*22.3O*	350 bp	4,800 bp	White		Fine
	*31.21O*	447 bp + 350 bp	3,400 + 3,600 bp	White		Thick

S36v	*18.1J*	350 bp	3,700 bp	White		Fine

S38v	*2.9F*	447 bp	4,000 bp + 4,200 bp		nv	

*nv, No installed velum.*

Interestingly, among the strains isolated in the velums, two clones (12.2O; 14.47O) sharing the same genotype (S9f) present in velums 12 and 14 had neither a long *FLO11* nor a deletion in *ICR1* (**Table 6**) and, as mentioned before, they did not have the typical *HaeIII* profile. However, they were able to form a velum. Although a very rare event, this is not surprising. Indeed, although the expression of *FLO11* has been shown to be the key event for biofilm formation, other genes, i.e., *FLO5, FLO9, FLO10* encoding Flo5p, Flo9p, and Flo10p confer cell–cell adhesion. Moreover, it cannot be excluded that *FLO11* expression and cell hydrophobicity could be linked to factors other than *ICR1* deletion or a long *FLO11* gene. Indeed, the regulation of *FLO11* is complex and depends on different specific pathways: the cAMP-protein kinase A (PKA) pathway; the mitogen-activated protein kinase (MAPK) pathway; and the TOR pathway (Braus et al., 2003; Vinod et al., 2008). Moreover, it has been shown that biofilm formation is also dependent on fatty acid biosynthesis (Zara et al., 2012). Fierro-Risco et al. (2013) also reported that the expression of stress-related genes (*SOD1, SOD2, HSP12*) could favor velum formation and thickness. More recently, Coi et al. (2017) demonstrated that flor yeasts possess specific *SFL1, RGA2* alleles that enhance flor formation. These results support the view that although *FLO11* polymorphism is an important characteristic of flor yeast and plays a key role in velum formation, other genes might be involved, and that the environment probably influences the nature of the velum.

To check the link between yeast flor phenotypes, especially the thickness of the velum and the polymorphism of *FLO11*, we compared the size of *ICR1* and *FLO11* with the velum characteristics. As shown in **Table 6**, there is no clear link between yeast phenotypes, especially the thickness of the velum and the polymorphism of *FLO11*.

Strains with a wild type *ICR1* ncRNA and a long Flo11p are expected to develop thin velum (Legras et al., 2014). However, in our study, *S. cerevisiae* clones classified in genotype S3v (11.8F, 20.1F, and 21.3F possessed a deletion in the *ICR1*nc RNA sequence but were isolated from thick velum (**Table 6**). On the other hand, S6 8.1J and S1 5.8F was isolated from thin velum but possessed a wild *ICR1* allele (**Table 6**). Strain 33.29O formed thick velum and possessed the deletion in its ICR1 promoter. These results show that the presence of the 111 bp deletion in the *ICR1* ncRNA was not always related to thin velum, as suggested previously (Legras et al., 2014). Our results support recent findings in which flor formation ability was shown to be variable in a flor strain with a specific deletion in the promoter of the *FLO11* gene (Kishkovskaia et al., 2017).

In these conditions, we wondered why some velums were thin and others thick. We hypothesized that the velum thickness might be related to the wine matrix. Indeed, according to our results, the same flor yeast could give different velums. For example, two isolates, namely 14.28O and 29.31O which were isolated in two different velums, one thin and one thick, presented the same inter delta pattern and the same *ICR1* and *FLO11* sequence. The same characteristics could be observed for the two strains 8.1J and 11.16F (**Table 6**). These results suggest that the same strain in two different matrixes can give different velums.

**FIGURE 4:**
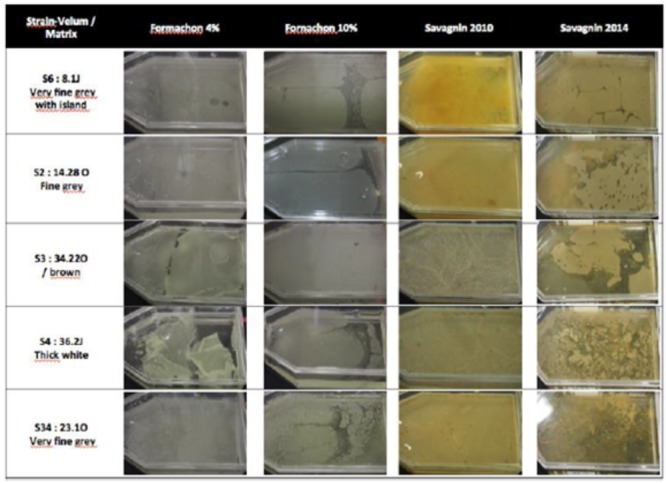
Structure and color of velum on different matrixes: grape, juice medium (Fornachon, 1953), synthetic [Fornachon 4% and 10% (v/v) ethanol] and two Savagnin wines (vintage 2010 and 2014) observed after 1 month. The inoculated strains are from different velums: S6: strain 8.1J from very fine gray velum with islands; S2: 14.2O strain from fine gray velum; S3: 32.22O from brown velum; S4: 36.2J strain from thick white velum and S34: 23.1O strain from very fine gray velum.

In order to confirm this, we inoculated different synthetic wines and Savagnin wine (vin jaune) with five different yeast strains 34.22O, 36.2J, 23.1O, 8.1J, 14.28O) possessing either a deletion in the promoter or a long *FLO11* gene or both (**Figure 4**). The color of the velum depended on both the medium and the age of the wine (**Figure 4**). While strain 34.22O gave a thick velum as expected (*ICR1* deletion), strain 8.1J sampled in a fine gray velum gave a thin pale gray velum in Fornachon medium [4 and 10% (v/v)] and a thick yellow/brown velum in a 2010 Savagnin wine and a thick white/cream velum in a 2014 Savagnin wine. Strain 14.28O gave a thick and thin velum in Fornachon and Savagnin, respectively. Strain 36.2J developed a thick pale gray velum in Fornachon 4%, a thin pale gray velum in Fornachon (10%) and a thick yellow velum in Savagnin 2010. Interestingly, the aspect of the velum differed as a function of the medium. While the velum developed with strain 34.22O was very smooth in Fornachon 4%, the velum presented wrinkles (ruffled pattern) in Savagnin 2010. Differences in velum aspects could also be observed for strain 36.2J when comparing all media, the major difference being between Fornachon and Savagnin Wine. These observations could be explained by the fact that biofilm formation is affected by nitrogen availability (Mauricio et al., 2001; Berlanga et al., 2006; Zara et al., 2011). Inositol availability has also been shown to influence biofilm formation (Zara et al., 2012). Thus, velum formation and velum characteristic are influenced by complex mechanisms involving both the genetic background of the yeast and wine composition.

## Conclusion

Our results show that Savagnin wine velums present very different characteristics never reported before in terms of color and morphology. Scanning electron microscopy analysis revealed remarkable differences in biofilm structure with distinct yeast morphologies and the presence of extracellular matrix. Despite all the differences observed, flor yeast genotyping demonstrated that most of the strains present in the velums belong to *S. cerevisiae* species and present the typical *HaeIII* ITS-RFLP flor yeast pattern. The genotyping analyses also demonstrate that a velum could be formed either of several different *S. cerevisiae* strains or one strain. Furthermore, a same strain could be present in velums presenting very different characteristics, supporting the view that wine composition plays a key role on velum characteristics. Our study also revealed population shifts during aging which reflects the fact that a strain could competitively displace another strain which could be linked to environmental changes during aging such as drastic temperature changes for example.

Finally, we confirmed in the present study the polymorphism of *FLO11* gene but we did not find any correlation between velum characteristic and *FLO11* polymorphism.

## Author Contributions

VD-V made all the laboratory experiments and made some wine sampling. HA sampled the wine, designed the experiments, and wrote the article. Interpretations were done by VD-V and HA.

## Conflict of Interest Statement

The authors declare that the research was conducted in the absence of any commercial or financial relationships that could be construed as a potential conflict of interest. The handling Editor declared a past co-authorship with one of the authors HA.

## Footnotes

1 http://www.ncbi.nlm.nih.gov/BLAST/
