# Corrigendum: Flor Yeast Diversity and Dynamics in Biologically Aged Wines

**DOI:** 10.3389/fmicb.2019.00363

**Published:** 2019-03-05

**Authors:** Vanessa David-Vaizant, Hervé Alexandre

**Affiliations:** ^1^AgroSup Dijon, PAM UMR A 02.102, Université Bourgogne Franche-Comté, Dijon, France; ^2^Equipe VAlMiS, Institut Universitaire de la Vigne et du Vin, Dijon, France

**Keywords:** flor yeast, biofilm, wine, *Saccharomyces cerevisiae*, scanning electron microscopy, *FLO11*, vin jaune

In the original article, we neglected to acknowledge Aline Bonnotte from Dimacell plateforme and Marie-Laure Leonard from ESIREM and ARCEN platform. The Acknowledgments section appears below: “We would like to acknowledge Aline Bonnotte from Dimacell plateforme and Marie-Laure Leonard from ESIREM and ARCEN platform, for providing microscopy images”.

The authors apologize for this error and state that this does not change the scientific conclusions of the article in any way. The original article has been updated.

