# Supplementary material for: Flor Yeast Diversity and Dynamics in Biologically Aged Wines
Source: Front Microbiol. 2018 Sep 25;9:2235. doi: 10.3389/fmicb.2018.02235 (PMC6167421; doi:10.3389/fmicb.2018.02235)
Supplement: Supplementary file 1 [file Table_1.DOC]

Table 1: Distribution (%) of *Saccharomyces cerevisiae* present in wine (W) at the beginning of the biological aging process or in velum (V) from two different wine estates, different seasons and different years of aging.
